# Multimodal Fusion of Environmental and Physiological Data for Real-World Personalised Comfort Modelling

**DOI:** 10.3390/s26102940

**Published:** 2026-05-07

**Authors:** Sothearak Heng, Ali Yavari

**Affiliations:** 1 School of Science, Computing and Emerging Technologies, Swinburne University of Technology, Melbourne, VIC 3122, Australia; 103798447@student.swin.edu.au; 2 6G Research and Innovation Lab, Swinburne University of Technology, Melbourne, VIC 3122, Australia

**Keywords:** personalised comfort modeling, heart rate variability, free-living physiological sensing, multimodal data fusion, indoor environmental quality, wearable sensors, machine learning, microclimate monitoring

## Abstract

People spend the majority of their lives within environments shaped by multiple interacting exposures, including thermal conditions, acoustic noise, lighting, and air quality, yet remain largely unaware of how these settings affect their comfort. Existing comfort research treats domains in isolation under controlled laboratory conditions, leaving real-world multi-domain effects on personal comfort underexplored. This paper proposes a unified Comfort Framework that fuses three practical data layers: macro-environmental conditions retrieved via location-based APIs, kinematic and micro-environmental context captured through smartphone sensors, and physiological responses recorded by a chest-worn ECG sensor. Binary comfort states are labelled in real time using a minimal-disruption lap-button protocol on a consumer smartwatch. We validate the pipeline through a single-subject pilot of 18 free-living sessions. Random Forest classification across 10 valid leave-one-session-out folds achieved an F1 macro of 0.456 ± 0.151, indicating that consumer wearable comfort prediction in unconstrained free-living conditions is more challenging than controlled chamber studies suggest. Descriptive statistics showed dataset-level differences between comfort states in wrist skin temperature (31.9 vs. 33.3 °C), heart rate (70.7 vs. 77.1 bpm), and RMSSD (42.1 vs. 34.3 ms), with overlap between classes consistent with the modest classification performance. SHAP analysis identified acoustic features, HRV features, and wrist temperature as the strongest comfort signals. The framework is architecturally designed to address all four IEQ domains, though this pilot empirically validated only thermal and acoustic signals.

## 1. Introduction

People often attribute fatigue, irritability, or reduced focus to stress or tiredness, unaware that their immediate environment may be the cause and that their body has already registered this discomfort before they have. Poor environmental conditions across four primary domains, thermal, acoustic, visual, and air quality, have been linked to Sick Building Syndrome, reduced productivity, impaired sleep quality, and broader health outcomes including cardiovascular and respiratory burden [1,2,3]. In office settings, sustained exposure to suboptimal indoor environmental quality has been associated with measurable decrements in cognitive performance and increased self-reported health complaints [4], while in residential settings poor environmental conditions disrupt sleep architecture and recovery [3]. Liu et al. [5] demonstrated that autonomic markers of thermal discomfort were measurably distinct between comfortable and uncomfortable conditions even when participants reported identical thermal sensations. Similarly, El Aarbaoui et al. [6] found that noise exposure triggered measurable autonomic nervous system responses at levels as low as 40 dB(A) in real-world settings, well below thresholds that register as consciously disturbing. These findings highlight the need for objective, continuous physiological monitoring to surface discomfort that subjective perception alone misses.

Existing comfort research addresses this need only partially. Studies have examined individual comfort domains in isolation, producing well-validated models such as PMV [7] for indoor thermal conditions and outdoor thermal stress indices for outdoor environments. However, these models rely on population-level averages, assume controlled steady-state environments, and do not capture the concurrent and interacting effects of multiple exposures on individual comfort. Yang et al. [8] demonstrated through a real-world field study that individual variability in thermal comfort is substantial, with PMV deviations of up to two units observed across subjects, directly motivating personalised over population-level models [9]. Machine learning has improved thermal comfort prediction substantially [8,10,11], but ML-driven physiological prediction has rarely extended beyond thermal conditions despite clear evidence that acoustic, lighting, and air quality exposures produce equally measurable autonomic responses [6,12,13]. Real-world deployment remains limited, with most physiological comfort studies confining participants to climate chambers or offices and leaving outdoor environments and transitions between contexts unaddressed [14].

A further methodological challenge specific to free-living physiological sensing has received insufficient attention in the comfort literature. Physiological signals in free-living conditions are heavily confounded by physical activity and posture, and consumer wearable sensor streams contain features that act as session or location identity proxies rather than comfort signals. Without explicit kinematic grounding and rigorous feature auditing, classification models risk learning environmental fingerprints rather than genuine comfort responses, producing apparent performance that does not generalise. Mishra et al. [15] established that contextual grounding is essential for reliable free-living physiological prediction in the stress detection domain, with Aqajari et al. [16] extending this finding using smartphone-based contextual data, but the implications for comfort sensing have not been systematically characterised.

We propose the Comfort Framework, a unified system that fuses three practical data layers to model personal comfort in real-world unconstrained environments. The first layer captures macro-environmental conditions via location-based APIs. The second captures kinematic and micro-environmental context through smartphone inertial, magnetic, and acoustic sensors. The third captures physiological responses through ECG-derived heart rate (HR) and heart rate variability (HRV) from a chest-worn sensor. Binary comfort states are labelled using a minimal-disruption lap-button protocol on a consumer smartwatch. Machine learning models trained on the fused feature set classify comfort states across indoor and outdoor settings, with SHAP analysis exposing individual feature contributions and data layer importance. The framework is architecturally designed to span all four IEQ domains, and the pilot was instrumented accordingly. However, not all domains contributed equally under the conditions of this pilot, and the practical constraints and their implications are discussed in Section 5.3 and Section 5.5.

The remainder of this paper is organised as follows. Section 2 reviews the related work. Section 3 describes the proposed framework. Section 4 presents the experimental setup and results, followed by Section 5, which discusses the findings. Section 6 concludes the paper.

## 2. Related Work

### 2.1. Multi-Domain Environmental Comfort

Environmental comfort is shaped by four primary domains, thermal, acoustic, visual, and air quality, whose effects are rarely experienced in isolation. Research has established that these domains interact nonlinearly, with overall comfort determined by whichever domain is most uncomfortable rather than any weighted average of domain scores [17,18]. Huang et al. [17] demonstrated this directly in an office study, finding that dissatisfaction with temperature or noise effectively vetoed overall environmental satisfaction regardless of lighting conditions, a pattern inconsistent with any linear composite index. Extending this to outdoor settings, Zhen et al. [19] showed that combined thermal and acoustic stress produced disproportionately greater discomfort than either stressor alone, confirming that cross-domain interactions are nonlinear and context-dependent. Despite this progress, multi-domain comfort studies have relied almost exclusively on subjective questionnaires and fixed-weight composite indices, with no physiological sensing and no machine learning prediction [18,19]. The most complete existing study, by Dai et al. [20], integrates temperature, CO_2_, PM_2.5_, noise, and lighting with continuous physiological monitoring and Random Forest prediction across commercial office environments, demonstrating that individual models consistently outperform general models. However, this study remains confined to indoor settings, does not capture kinematic context, and cannot separate activity-driven physiological changes from genuine environmental stress responses. No existing study integrates all four comfort domains with physiological sensing, machine learning, and real-world outdoor deployment simultaneously.

### 2.2. Physiological Indicators of Comfort

Physiological signals, particularly HR and HRV, provide an objective, continuous window into how environmental conditions affect the body, often before conscious discomfort is registered. Across thermal research, Liu et al. [5] showed that HRV shifted measurably between comfortable and uncomfortable thermal conditions even when participants reported identical sensations, establishing that physiological signals detect discomfort that subjective reporting misses entirely. Ibrahim et al. [21] further confirmed a nonlinear quadratic relationship between HR and PMV, with HR elevating consistently as conditions moved away from thermal neutrality in either direction. The same autonomic pathway responds to other environmental domains. Petrowski et al. [12] demonstrated that spectral light composition alone shifted autonomic balance, with blue light increasing sympathetic activity and red light enhancing parasympathetic tone. El Aarbaoui et al. [6] measured physiological responses to noise in genuine free-living conditions across 75 participants, finding sympathetic activation detectable at levels as low as 40 dB(A), confirming that physiological signals remain meaningful outside controlled settings. Cakmak et al. [13] demonstrated that particulate matter exposure increased maximum HR and reduced HRV in ambulatory subjects, extending this evidence to air quality. Collectively, these studies establish that HR and HRV carry comfort-relevant information across all four environmental domains. However, evidence remains fragmented across isolated domain studies conducted predominantly in controlled laboratory settings, with no study integrating physiological signals across all four domains simultaneously in real-world conditions.

### 2.3. ML-Driven Comfort and Stress Prediction

Machine learning has substantially advanced personalised comfort prediction, though almost exclusively within the thermal domain. Chaudhuri et al. [10] demonstrated that a Support Vector Machine (SVM) trained on wrist pulse rate and skin temperature could predict personal thermal comfort with approximately 97% accuracy in a climate chamber, establishing that wearable physiological signals contain sufficient information for comfort classification. Sahoh et al. [22] compared EEG, HRV, and EDA as biomarkers for thermal comfort prediction using deep learning, finding that HRV offered the best balance between predictive performance and computational efficiency for wearable deployment. Kim et al. [11] showed that combining multiple physiological signals achieved 79.7% accuracy with LightGBM, with SHAP analysis revealing that PPG-derived HRV features dominated prediction, validating HRV as the primary physiological signal for comfort modelling. Yang et al. [8] demonstrated through a real-world field study that individual variability in thermal comfort is substantial, with PMV deviations of up to two units observed across subjects, directly motivating personalised over population-level models. Despite these advances, ML comfort prediction has not extended beyond thermal conditions, and most studies remain confined to controlled indoor settings. A more mature body of work in ML-driven stress detection has demonstrated a critical methodological insight directly applicable to comfort modelling. Mishra et al. [15] showed in a free-living study that physiological features alone achieved an F1 score of 0.502, barely above chance, while adding contextual features including activity type and time of day improved this to 0.769. Notably, context alone outperformed physiology alone, establishing that physiological signals without contextual grounding are unreliable in the wild. Aqajari et al. [16] reinforced this through smartphone-based contextual data, improving stress classification from 56% to 70% F1 with no additional burden on participants. Since stress and comfort share the same underlying autonomic mechanism, this methodological lesson transfers directly: physiological comfort prediction in free-living conditions requires explicit kinematic and contextual grounding to be meaningful. The present study applies this principle to comfort modelling across all four environmental domains.

## 3. Proposed Framework

This section provides a description of the proposed framework.

### 3.1. System Overview

The Comfort Framework fuses three distinct data layers to model personal comfort in real-world, unconstrained environments. The first layer captures macro-environmental conditions through location-based APIs, providing ambient temperature, humidity, wind speed, solar radiation, and air quality indices without requiring personal environmental hardware. The second layer captures kinematic and micro-environmental context through smartphone inertial, magnetic, acoustic, and light sensors, recording physical activity, posture, ambient sound level, and immediate environmental context. The third layer captures physiological responses through a chest-worn ECG sensor, providing heart rate and heart rate variability at millisecond resolution. The framework is architecturally designed to span the four major IEQ domains of thermal, acoustic, visual, and air quality, with each layer contributing signal toward different domains. Together, these layers provide explicit kinematic context to support the interpretation of physiological signals in free-living conditions, where activity, posture, and movement substantially confound autonomic measurements.

**Figure 1:**
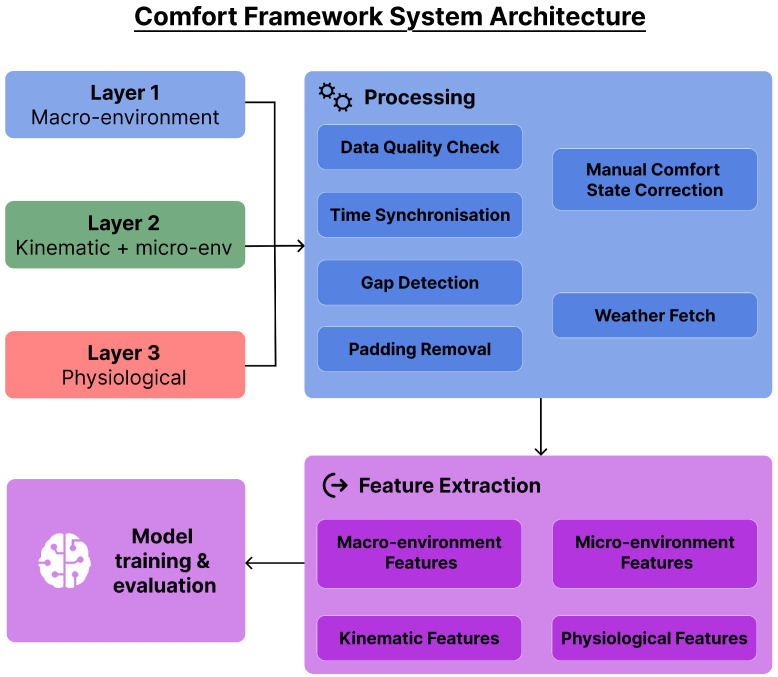
System architecture of the Comfort Framework. Three data layers feed into a preprocessing pipeline covering data quality assessment, time synchronisation, gap detection, and padding removal, followed by feature extraction and model training.

Binary comfort classification, comfortable versus uncomfortable, is the primary prediction target. This binary formulation was deliberately chosen to minimise disruption to natural behaviour during data collection, as finer-grained comfort scales require more frequent and cognitively demanding self-reports that would themselves alter the comfort state being measured. The overall system architecture is illustrated in Figure 1.

### 3.2. Instrumentation

**Figure 2:**
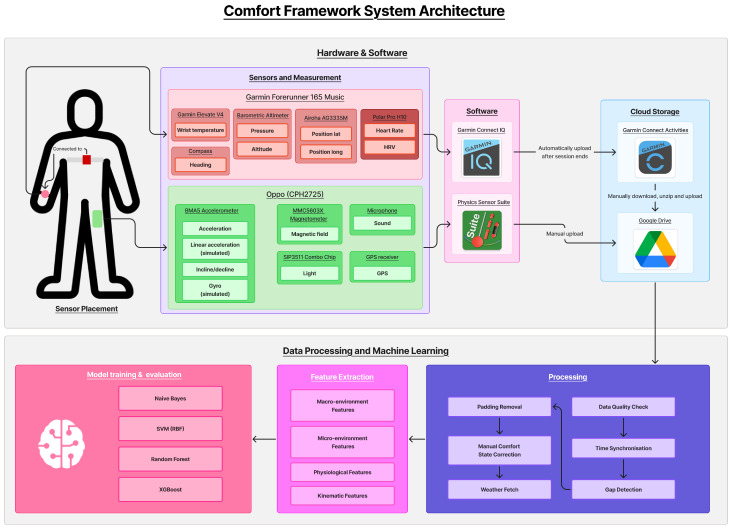
Complete system architecture of the Comfort Framework. Top: hardware and software components, showing the Garmin Forerunner 165 Music recording physiological and kinematic data from the Polar H10 chest strap via Bluetooth, and the OPPO CPH2725 smartphone capturing kinematic, acoustic, magnetic, and light sensor streams via Physics Toolbox Sensor Suite. Session data is exported automatically from the Garmin sensor to Garmin Connect Activities and uploaded manually from the OPPO to Google Drive. Bottom: the data processing and machine learning pipeline, covering preprocessing, feature extraction across four data layers, and binary comfort classification using four machine learning models.

Figure 2 illustrates the complete system architecture and instrumentation used throughout all data collection sessions.

#### 3.2.1. Physiological Sensing

Heart rate and RR intervals are recorded using the Polar H10 chest strap connected via Bluetooth to the Garmin Forerunner 165, which also records heart rate, a 25 Hz triaxial accelerometer, barometric pressure, altitude, GPS coordinates, and heading at one-second intervals in its record messages. Millisecond resolution is appropriate for cardiac monitoring because the human heart beats at roughly 40 to 180 beats per minute under normal conditions, meaning the time between successive heartbeats ranges from approximately 333 ms to 1500 ms [23]. HRV features such as RMSSD and pNN50 are derived from the precise timing differences between these beats, differences that can be as small as a few milliseconds, making millisecond resolution the minimum necessary to capture meaningful autonomic variation. Coarser sampling rates typical of optical PPG wearables lose this fine-grained temporal information and produce unreliable HRV estimates [22]. The Polar H10 was therefore selected as the primary physiological device because ECG-derived RR intervals represent the gold standard for HRV analysis, providing the signal quality necessary for reliable time-domain, frequency-domain, and nonlinear HRV feature extraction. Unlike PPG-based wearables, ECG measurement is not susceptible to optical motion artifacts, making it particularly suited to free-living data collection across diverse activity contexts. Within the thermal domain specifically, the framework adopts a complementary approach to traditional environmental measurement. Rather than directly measuring operative temperature components such as mean radiant temperature and air velocity, which require specialised environmental sensors, the framework captures the body’s integrated thermal response through wrist skin temperature and autonomic markers. The human body responds to the combined effect of all thermal environmental components simultaneously, providing a physiologically grounded thermal signal that does not depend on any single environmental measurement.

#### 3.2.2. Kinematic and Micro-Environmental Sensing

Kinematic and micro-environmental context data were captured using an OPPO CPH2725 Android smartphone running the Physics Toolbox Sensor Suite application with battery optimisation disabled and developer mode enabled to ensure continuous full-rate sensor logging. The following sensor streams are recorded simultaneously: triaxial accelerometer capturing linear acceleration, triaxial gyroscope capturing rotational movement, gravity sensor capturing device orientation, magnetometer capturing ambient magnetic field strength, microphone capturing sound level in decibels, and luxometer capturing ambient light intensity. These signals collectively characterise the participant’s physical activity, posture, and immediate micro-environment, variables that both confound and contextualise physiological responses to environmental stressors. A power bank was used throughout each session to prevent sensor dropout.

The smartphone was carried in the right trouser pocket for all sessions with the screen facing outwards and the camera end pointing downward. This deployment position was selected as a deliberate design decision to maintain consistent sensor placement across all sessions and to minimise disruption to natural behaviour during data collection. Alternative deployments such as handheld carrying or chest-mounted positioning would have introduced session-to-session variation in sensor orientation, inadvertent screen interactions, and behaviour modification as attention is directed toward the device rather than the natural activity being recorded, all of which would compromise the ecological validity that the framework is designed to preserve.

A consequence of pocket deployment is that the luxometer measures pocket openness rather than ambient environmental light, and the feature was therefore excluded from the modelling feature set. This represents a trade-off between visual domain capture and overall sensor consistency, with the framework prioritising the latter. The pipeline is architecturally designed to incorporate ambient light data, and a wrist-worn or clip-on light sensor, or a revised smartphone deployment position such as a shirt pocket mount, would allow the visual domain to be captured in future deployments without compromising the frictionless collection protocol. Sound level and magnetic field features were retained as both signals remained meaningful in the pocket deployment context.

#### 3.2.3. Comfort State Labelling

Comfort state labels were captured using the Garmin Forerunner 165 Music smartwatch via a custom Garmin Connect IQ application developed for this study. The application presents a pre-session checklist confirming GPS signal quality and Polar H10 connection status by detecting the presence of R-R inter-beat intervals, which are transmitted exclusively by the ECG chest strap and not by the wrist optical sensor. Comfort state was encoded through the lap function: even lap indices represent a comfortable state and odd lap indices represent an uncomfortable state. A comfortable state was assumed at session start. This approach was selected to minimise disruption to natural behaviour and cognitive load during data collection, consistent with ecological momentary assessment principles [15]. Where a lap press was missed during a session, corrections were applied during preprocessing by manually overriding the state of a specific lap index or inserting additional lap boundaries at known transition times, verified against handwritten session notes. The Garmin additionally recorded ambient wrist temperature throughout each session, providing a localised thermal micro-environment reading that reflects the individual’s actual thermal exposure rather than regional meteorological estimates.

The Garmin Forerunner 165 stores lap events as a sequence of start timestamps rather than start–end pairs. Lap durations are therefore computed from consecutive start times: the duration of lap *i* equals start_time[i+1]− start_time[i], with the final lap running from its start time to the session end. This approach was verified to be consistent across all sessions.

#### 3.2.4. Macro-Environmental Data

Macro-environmental conditions were retrieved retrospectively for each session using the Open-Meteo weather and air quality APIs. This layer captures regional outdoor environmental conditions at the session location rather than localised indoor measurements, providing ambient meteorological and air quality context without requiring personal environmental sensors. Given the GPS coordinate and session timestamp from the session log, the archive API is queried as the primary source. For sessions recorded within the archive lag window of approximately five days, the forecast API with historical access is used as a fallback. The API returns hourly resolved values averaged across the session window.

A broad set of variables is retrieved at this stage rather than only the variables anticipated for modelling. This design decision keeps the raw data collection layer decoupled from the modelling layer; the variables that prove uninformative or redundant can be excluded during feature selection without requiring a re-fetch of the raw data. Weather variables retrieved include: temperature, apparent temperature, relative humidity, dew point, wet bulb temperature, vapour pressure deficit, precipitation, surface pressure, cloud cover, wind speed, wind direction, wind gusts, shortwave radiation, direct radiation, diffuse radiation, and evapotranspiration. Air quality variables include: PM_2.5_, PM_10_, carbon monoxide, nitrogen dioxide, ozone, UV index, dust, ammonia, alder pollen, birch pollen, grass pollen, European AQI, and US AQI. For the Melbourne pilot dataset, ammonia, alder pollen, birch pollen, and grass pollen returned null values across all sessions and were excluded from the feature set. All other retrieved variables were carried forward as candidate features for the modelling stage.

The framework recognises a distinction between regional outdoor environmental data captured by this layer and localised indoor environmental conditions including CO_2_ accumulation, particulate matter, volatile organic compounds, and odours, which can substantially affect occupant comfort but are not resolvable from regional API estimates. Capturing localised indoor environmental data would require a personal wearable air quality monitor such as the Atmotube Pro, which measures VOCs, PM_1_, PM_2.5_, PM_10_, temperature and humidity in a compact form factor consistent with the framework’s consumer-grade hardware design principle. Such a device would integrate into the existing pipeline as an additional data layer without requiring modifications to the feature extraction or modelling architecture, and is identified as a hardware addition for future deployments seeking to capture indoor air quality variation.

#### 3.2.5. Timestamp Synchronisation

Each session involves two independent device clocks: the Garmin Forerunner 165, which simultaneously records the Polar H10 RR intervals via Bluetooth and comfort state labels via the lap function, and the OPPO running Physics Toolbox capturing kinematic and micro-environmental sensor streams. To achieve precise temporal alignment between these two streams, a three arm-swing gesture was performed at the session start, and where possible repeated at the session end. The arm swings produce a distinctive high-magnitude spike in the OPPO accelerometer and a simultaneous disturbance in the Garmin accelerometer stream, providing a shared reference event visible on both devices.

Alignment was performed manually: the first 20 s of each session were plotted for both the OPPO and Garmin accelerometer streams, and the elapsed time of the last swing is identified visually on each device. These two times are entered as sync anchor points, and the OPPO elapsed-time axis is shifted to the Garmin UTC clock using the formula

(1) $$t_{\mathrm{OPPO},\;\mathrm{UTC}}=t_{\mathrm{Garmin},\;\mathrm{UTC}}-\left(t_{\mathrm{Garmin},\;\mathrm{sync}}-t_{\mathrm{OPPO},\;\mathrm{sync}}\right)$$

where $t_{\mathrm{Garmin},\;\mathrm{sync}}$ and $t_{\mathrm{OPPO},\;\mathrm{sync}}$ are the elapsed times of the sync gesture on each device, respectively. Sessions where the sync gesture is absent or ambiguous are flagged for manual review. Garmin lap timestamps serve as the reference for comfort state label alignment.

Garmin record messages are timestamped as Melbourne local time strings with no date component. These are converted to the aligned timeline by combining each time string with the session date derived from the sync anchor, then computing elapsed seconds from the sync point. A midnight crossover guard handles sessions that span midnight; one session began at 23:26 and continued past midnight, requiring post-midnight records to be shifted forward one day before elapsed time computation.

### 3.3. Data Collection Protocol

Data collection was conducted as a single-subject pilot study to validate the complete framework pipeline in genuine free-living conditions before scaling to a larger cohort. This approach follows the personalised comfort modelling paradigm established by Kim et al. [9], which demonstrates that individual comfort models outperform population-level models, a paradigm that requires per-person data collection at the scale this pilot is designed to enable.

Sessions were conducted during normal daily activities without deliberate environmental manipulation. Each session varies in duration but is a minimum of 10 min. A comfortable state is assumed at session start, with lap button presses on the Garmin marking comfort state transitions throughout the session. The first and last 90 s of each session were removed as padding to eliminate transient physiological responses at session boundaries.

For each session, a session log was maintained recording the session date, start time, location name, GPS coordinates, indoor/outdoor classification, and any observational notes. GPS coordinates from the session log are used by the pipeline to retrieve macro-environmental data retrospectively from the Open-Meteo API.

Sessions were conducted across a range of environments encountered during normal daily activity, including indoor home, work, library, café, and transport settings, alongside outdoor walking routes. The pilot dataset comprises 18 sessions collected over multiple weeks, spanning indoor and outdoor contexts, comfortable and uncomfortable conditions, and morning, afternoon, and evening periods, providing sufficient variety to validate the complete pipeline.

### 3.4. Preprocessing and Feature Engineering

#### 3.4.1. Data Quality Assessment

Prior to alignment and feature extraction, each session underwent a data quality check. Sessions were excluded from all subsequent stages if required files were missing. For sessions where files were present, the OPPO time series was scanned for the first inter-sample gap exceeding 5 s, which is treated as a data loss event caused by Android OS sensor throttling when the phone screen turns off. Sessions with no significant gap proceeded normally: 90 s of padding were applied at both the start and end of the usable window. Sessions with a significant gap were trimmed to the last good sample before the gap with 90 s of start padding only; end padding was omitted because the data ends mid-session without the subject’s awareness of the session ending, meaning there are no session-end behavioural artifacts to remove.

#### 3.4.2. Timestamp Alignment and Windowing

Automatic sync detection was attempted across three approaches before manual identification was adopted. The first approach used a quiet threshold to detect the end of the sync burst, but failed for outdoor walking sessions where activity immediately following the sync swings kept the signal above the threshold. A relative quiet threshold adapting to session peak magnitude improved this but remained unreliable. The second approach used zero-crossing rate transitions to distinguish slow arm swings from rapid walking oscillations, a physically meaningful signal that worked for most sessions but failed for one where the transition was not detected within the search window. The third approach used peak detection within a 15-s window, which worked for most sessions but still required session-specific threshold tuning. After three rounds of automated approaches each requiring manual exception handling, fully manual sync point identification was adopted. Visual inspection of the first 20 s of each session at 0.5-s tick resolution takes approximately two minutes per session and eliminates all sync detection errors. The manually identified sync times are stored as ground truth in MANUAL_SYNC_POINTS.

Following sync point identification, the OPPO elapsed-time axis is shifted to the Garmin UTC clock using the formula described in Section 3. The session end is set to the earlier of the last OPPO sample and the last Garmin record message, preventing dead recording time from being included if one device stopped before the other. For sessions with no data gap, 90 s of padding are then removed from both the start and end of the usable window. For sessions with a data gap, only start padding is applied and the session is trimmed to the last good sample before the gap.

#### 3.4.3. HRV Feature Extraction

HRV features are extracted from the Polar H10 RR interval stream using non-overlapping 60-s windows. The 60-s window length sits below the 5-min short-term recording duration recommended by the Task Force standards for conventional HRV analysis [23], and falls within the ultra-short-term HRV regime that has received substantial methodological attention in the recent wearable sensing literature. Within this regime, time-domain measures dominated by short-term beat-to-beat variation, particularly RMSSD and pNN50, retain reasonable reliability at 60-s resolution, while measures requiring longer observation windows, particularly LF power and SDNN, are known to be substantially less reliable [24,25]. The 60-s window length was selected as a deliberate trade-off between HRV precision and the temporal resolution required to track comfort state transitions in free-living conditions, where a 5-min window would span multiple lap-button comfort labels and confound transition dynamics. The implications of this trade-off for feature interpretation are addressed in Section 5.5. Non-overlapping windows are used rather than overlapping to avoid temporal correlation between adjacent windows, which would violate the independence assumptions of the classifiers. Windows with fewer than 30 beats after artifact removal are discarded.

Artifact rejection is applied before windowing. RR intervals outside the physiologically plausible range of 333 ms to 1500 ms are removed. The lower bound corresponds to 180 beats per minute and excludes ectopic beats and motion artifacts. The upper bound corresponds to 40 beats per minute and excludes missed beats and signal loss. A simple threshold approach was used rather than adaptive local-mean methods because adaptive rejection removed too many beats during genuine cardiac activity changes in ambulatory conditions, consistent with the Task Force recommendation for ambulatory HRV monitoring [23].

Beat timestamps are reconstructed by anchoring the first beat to the start of the usable session window and cumulatively summing RR intervals in seconds. This is valid because the processing pipeline already trimmed garmin_hrv.csv to the usable window, ensuring the first beat in the file is the first beat after padding.

Time-domain features extracted per window include: mean RR interval, SDNN, RMSSD, SDSD, pNN50, and mean heart rate derived from the mean RR interval. Frequency-domain features are computed using the Lomb–Scargle periodogram rather than the Fast Fourier Transform. RR intervals are by definition non-uniformly sampled as heartbeats do not arrive at regular intervals and the Lomb–Scargle periodogram computes spectral power directly from irregular timestamps without interpolation, avoiding the spectral leakage introduced by FFT-based methods on interpolated RR series [23]. LF power (0.04–0.15 Hz) reflects mixed sympathetic and parasympathetic modulation. HF power (0.15–0.40 Hz) reflects parasympathetic vagal tone entrained by respiratory sinus arrhythmia. The LF/HF ratio captures sympathovagal balance, with values above 2 indicating sympathetic dominance. Total power integrates across both bands. Very low frequency power is not computed because 60-s windows contain insufficient data points to resolve the VLF band reliably. Nonlinear features include SD1 and SD2 from the Poincaré plot, the SD1/SD2 ratio, and sample entropy computed using the Richman and Moorman algorithm with the embedding dimension m=2 and tolerance r=0.2×SD of the RR series [26].

#### 3.4.4. Kinematic and Physiological Record Feature Extraction

OPPO kinematic features are extracted at 10 Hz (600 samples per window, 480 minimum). Windows below the minimum sample threshold are discarded, implicitly handling data gap periods which contribute near-zero samples. From the triaxial accelerometer, the mean, standard deviation, minimum, and maximum of the vector magnitude $\left|a\right|=\sqrt{a_{x}^{2}+a_{y}^{2}+a_{z}^{2}}$ are computed, alongside the mean and standard deviation of each individual axis. Jerk is computed as $j_{i}={\left||a\right|}_{i+1}-{\left|a\right|}_{i}|/\Delta t_{i}$ and summarised as mean, standard deviation, and maximum per window. From the gyroscope, magnitude mean, standard deviation, and maximum are extracted alongside per-axis mean and standard deviation. Gravity component means capture device orientation and posture context. Illuminance features were extracted but subsequently excluded from the modelling feature set, as the trouser pocket deployment meant the luxometer measured pocket openness rather than ambient light, capturing activity patterns already represented by accelerometer features.

From the Garmin 1 Hz record stream, heart rate statistics (mean, SD, min, max) and heart rate reserve are extracted. Heart rate reserve normalises HR to the participant’s personal physiological range:

(2) $${\mathrm{HR}}_{\mathrm{reserve}}=\frac{\bar{HR}-{\mathrm{HR}}_{\mathrm{rest}}}{{\mathrm{HR}}_{\max}-{\mathrm{HR}}_{\mathrm{rest}}}\times 100$$

where ${\mathrm{HR}}_{\max}=203$ bpm from the participant’s Garmin profile and ${\mathrm{HR}}_{\mathrm{rest}}=60$ bpm is a standard approximation for a fit young male. Wrist temperature mean, standard deviation, and rate of change are extracted. The rate of change captures thermal transition dynamics that the mean alone cannot reflect; a participant moving between environments shows an elevated rate of change even before the mean temperature stabilises within the window. The Garmin wrist accelerometer operates at 25 Hz and is stored as packed JSON arrays (25 values per 1-s row). These are unpacked to produce approximately 1500 samples per 60-s window, from which magnitude mean, standard deviation, and maximum are extracted. Heading circular variance is computed using the mean resultant vector length to handle the 0/360 degree boundary correctly, capturing how much the participant changed direction during the window.

#### 3.4.5. Feature Vector Construction

Following preprocessing, features are extracted from each data layer and combined into a unified feature vector per window, as summarised in Table 1. Within-subject normalisation is applied to physiological and kinematic features by subtracting the session-level baseline and dividing by the standard deviation, ensuring the model learns deviations from personal baseline rather than absolute values. This is appropriate for signals where the meaningful information is the deviation from the individual’s resting state.

Acoustic features are retained in absolute form rather than normalised within session. The decibel level captured by a consistently positioned smartphone microphone represents genuine acoustic exposure that is directly comparable across sessions, and within-session normalisation would remove the absolute level information that distinguishes a quiet environment from a noisy one. Retaining absolute sound values allows the model to learn the relationship between acoustic exposure level and comfort state across environments rather than only capturing within-session acoustic fluctuation. Within-session normalisation is retained for magnetometer features as the absolute magnetic field magnitude varies substantially with location in ways that do not reflect comfort state.

A subset of extracted features were excluded from the modelling feature set for two distinct reasons. First, features with technical limitations were excluded prior to modelling: macro-environmental variables retrieved from the Open-Meteo API varied between sessions only and acted as session identity proxies; light features were excluded as the trouser pocket deployment captured pocket openness rather than ambient light; pressure and altitude rate features varied primarily between sessions; gps_speed_mean was 87.6% missing; gyro_mag_min was near-constant; n_beats was identified as a data quality indicator rather than a comfort feature; and contextual session proxies (is_outdoor, time-of-day encodings) were excluded to prevent the model from learning session identity directly. Second, following an empirical review of feature contributions, additional features were identified as session and location identity proxies and excluded from the final feature set. Altitude varies between locations but does not reflect comfort state within a session. Phone gravity components (gFx, gFy, gFz) capture phone orientation in the pocket, which varies with posture but does not directly reflect comfort. Per-axis magnetometer values (mag_x, mag_y, mag_z) capture the direction of the magnetic field relative to phone orientation, which is fixed by location and phone position rather than reflecting ambient field strength. Magnetometer magnitude features (mag_mag_mean, mag_mag_sd, mag_mag_min, mag_mag_max) were retained as they are rotation-invariant and reflect the genuine ambient magnetic environment. Heading circular variance was excluded as direction change captures activity context rather than comfort. The implications of these exclusions for classification performance are reported in Section 4.

### 3.5. Machine Learning Pipeline

#### 3.5.1. Models

Comfort prediction is formulated as a binary classification task. Four classifiers are evaluated. Naive Bayes serves as a probabilistic baseline. Support Vector Machine with a radial basis function kernel serves as a discriminative baseline. Random Forest is selected as the ensemble model based on its consistent strong performance in physiological and contextual comfort and stress prediction tasks [15,16,20]. XGBoost is evaluated as a gradient boosting alternative. The pipeline supports class imbalance handling through synthetic minority oversampling technique (SMOTE) applied to the training set only, combined with adjusted class weights in all classifiers. While these mechanisms are integrated into a framework for deployments where class imbalance is severe, the pilot dataset achieved approximately balanced class representation through deliberate session selection, and imbalance handling had limited impact on overall classification performance.

#### 3.5.2. Evaluation and Interpretability

Model evaluation uses leave-one-session-out (LOSO) cross-validation. In each fold, one session is held out as the test set and the model is trained on the remaining sessions. Primary evaluation metrics are F1 macro and F1 for the uncomfortable class. F1 macro weights both classes equally regardless of frequency and is the appropriate primary metric under class imbalance. F1 for the uncomfortable class is additionally reported because failing to detect discomfort is the more consequential error in a comfort monitoring application. Precision, recall, and area under the ROC curve are reported as secondary metrics. SHAP values are computed for the best-performing model trained on the full dataset to produce stable feature importance estimates across all sessions.

## 4. Experiments

### 4.1. Experimental Setup

Data collection was conducted by one of the authors across 18 sessions in Melbourne, Australia. Author characteristics are: age 22, height 168 cm, weight 70 kg, male. Sessions spanned indoor and outdoor environments including home, warehouse, library, café, train, and outdoor walking routes. Sessions were distributed across morning, afternoon, and evening periods. The dataset comprises 418 labelled 60-s windows across all sessions, summarised in Table 2.

As shown in Table 3 the descriptive statistics provide empirical context for the classification and SHAP results. Wrist temperature and heart rate show the clearest between-state separation, with comfortable windows associated with lower wrist temperature (31.9 vs. 33.3 °C) and lower heart rate (70.7 vs. 77.1 bpm), and RMSSD elevated under comfortable conditions (42.1 vs. 34.3 ms) consistent with stronger parasympathetic activity reported in the thermal comfort literature [5]. Acoustic features show smaller global between-state differences (sound mean 78.0 vs. 77.4 dB; sound SD 5.9 vs. 4.8 dB), indicating that the predictive contribution of acoustic features identified in the SHAP analysis arises from within-session and interaction-level structure rather than from large global mean differences. Phone accelerometer magnitude differs at the dataset level (0.42 vs. 0.78 m/s^2^), but as discussed in Section 5.2 this difference is driven primarily by a small number of high-movement sessions rather than a within-session activity–comfort coupling.

A notable structural characteristic of the dataset is that 8 of the 18 sessions are single-class, either entirely comfortable or entirely uncomfortable. This reflects the real-world nature of the data collection: some environments were consistently comfortable or uncomfortable throughout the session with no state transitions. As a consequence, only 10 of the 18 LOSO folds produced test sets with both comfort classes and could be used for evaluation. The remaining eight sessions still contribute to training in folds where they are not the held-out test set, so they are not entirely lost to the modelling. The class distribution across all windows is approximately balanced (53.3% comfortable, 46.7% uncomfortable), reflecting deliberate session selection across both naturalistic and varied indoor and outdoor environments.

### 4.2. Evaluation Metrics and Cross-Validation

Leave-one-session-out (LOSO) cross-validation was adopted as the evaluation strategy. In each fold, one session is held out as the test set and the model is trained on the remaining 17 sessions. This approach ensures that the model is evaluated on data from a session it has not seen during training, providing an honest estimate of generalisation to new sessions and contexts.

As described in Section 4, 8 of the 18 sessions are single-class. These sessions cannot serve as valid test folds because no meaningful binary classification metrics can be computed when the test set contains only one class, and results are therefore reported across the 10 valid folds as mean ± standard deviation.

F1 macro is reported as the primary metric as it weights both classes equally regardless of their frequency. F1 for the uncomfortable class is additionally reported because failing to detect genuine discomfort is the more consequential error for a real-world comfort monitoring application. Precision, recall, and AUC-ROC are reported as secondary metrics.

### 4.3. Results and Analysis

#### 4.3.1. Classification Performance

Table 4 presents the cross-validation results across the 10 valid folds. Random Forest achieved the highest F1 macro (0.456 ± 0.151) and is therefore treated as the best-performing model for subsequent SHAP analysis. The four classifiers produced F1 macro values between 0.37 and 0.46, with substantial standard deviations relative to the differences between models, indicating that the choice of classifier is not the dominant factor in comfort prediction performance on this dataset. The F1 macro range falls substantially below performance reported in controlled chamber studies, where F1 values above 0.80 are commonly reported [10,11], indicating that comfort classification in this free-living dataset is more challenging than controlled deployments suggest.

The large standard deviations across folds reflect genuine fold-to-fold variance rather than measurement noise. Test set sizes range from 8 to 36 windows across the 10 valid folds, and class balance varies substantially between folds, with some folds containing as few as 4 windows of the minority class. A single correct or incorrect prediction in small or imbalanced folds shifts F1 by 10 to 15 percentage points. The Random Forest F1 macro range across individual folds spans from 0.241 to 0.733, illustrating that classification performance varies considerably depending on how well the held-out session resembles the training data. The high variance is an expected consequence of the small dataset size and the diversity of free-living environments captured, and is discussed further in Section 5.

**Figure 3:**
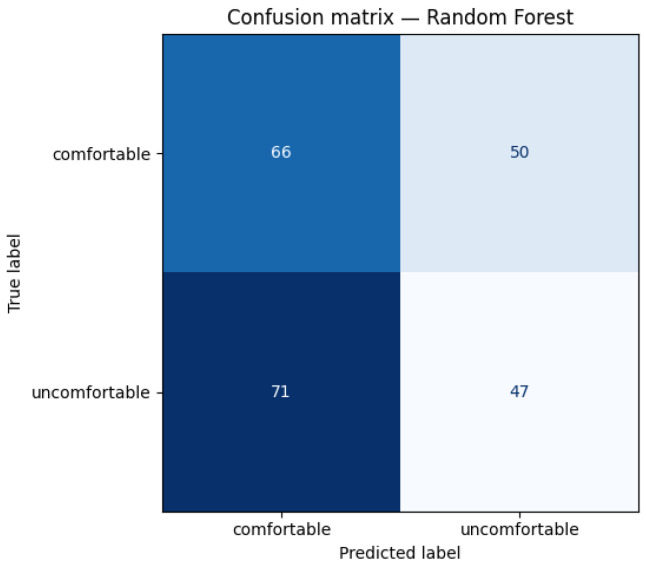
Confusion matrix for Random Forest across all 10 valid LOSO folds combined (234 test windows: 116 comfortable, 118 uncomfortable). Overall accuracy is 48.3%.

Figure 3 shows the combined confusion matrix for Random Forest across all 10 valid folds (234 total test windows).

The confusion matrix reveals that the model fails to reliably detect uncomfortable windows in this dataset. Of 118 uncomfortable windows in the test set, only 47 are correctly identified, with 71 misclassified as comfortable. This false negative rate of 60% is the most consequential error type for a real-world comfort monitoring application, as undetected discomfort cannot trigger any corrective response. The model’s predictions are approximately balanced between classes (137 predicted comfortable, 97 predicted uncomfortable), but the predictions do not align well with the true labels in either direction, indicating the underlying signal is weak rather than the model being biased toward one class. This performance reflects the genuine difficulty of free-living comfort prediction and is interpreted in detail in Section 5.

#### 4.3.2. Feature Importance Analysis

**Figure 4:**
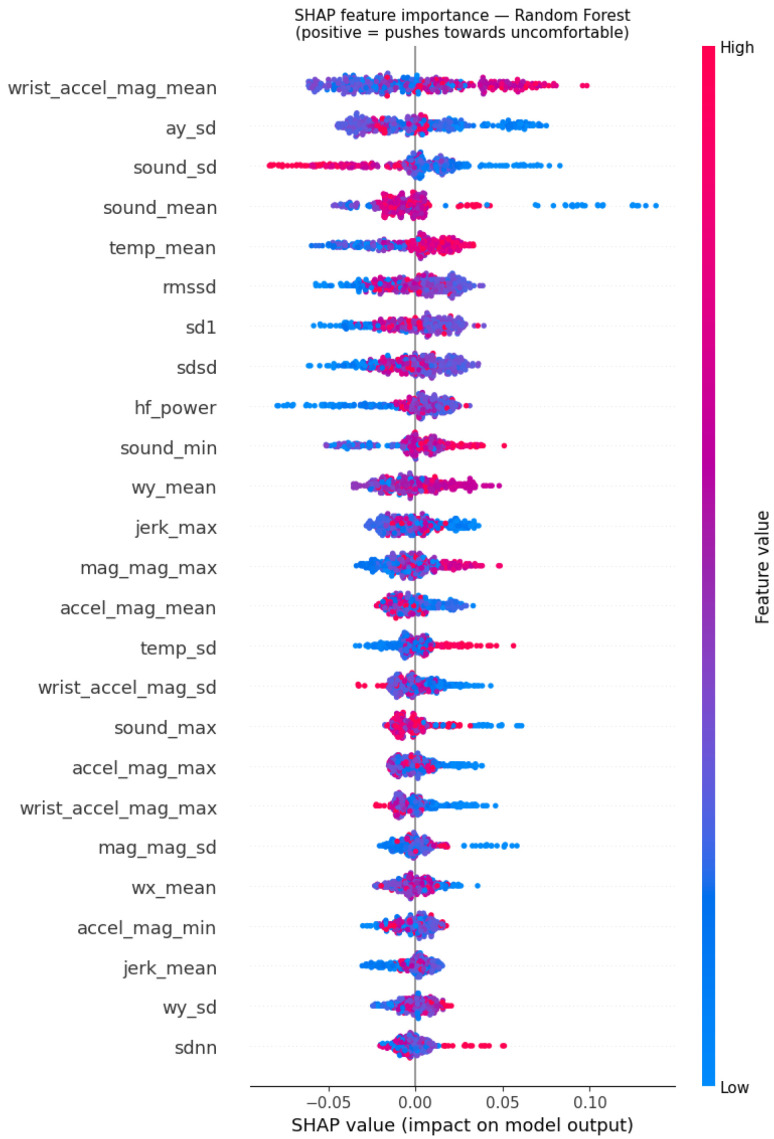
SHAP beeswarm plot for Random Forest trained on the full 418-window dataset. Each point represents one window. Colour indicates feature value (red = high, blue = low). Points to the right push predictions toward uncomfortable; points to the left push toward comfortable. Features are ranked by mean absolute SHAP value. The distribution of points reveals both the direction and consistency of each feature’s contribution across windows.

SHAP values were computed for Random Forest trained on the full 418-window dataset. Figure 4 shows the beeswarm plot of SHAP values for the top 25 features, and Table 5 lists the top 10 features by mean absolute SHAP value.

The top 10 features comprise four acoustic features (sound_sd, sound_mean, sound_min, with sound_max in nearby ranks), four HRV features (rmssd, sd1, sdsd, hf_power), one thermal feature (temp_mean), and two kinematic context features (wrist_accel_mag_mean, ay_sd). This distribution shows that genuine comfort signals, acoustic exposure, physiological response, and thermal state together account for the majority of the top features, with kinematic features serving as activity context.

Table 6 summarises mean absolute SHAP contributions aggregated by data layer.

The acoustic layer contributes the highest mean SHAP per feature, reflecting the dominant role of sound exposure in driving comfort predictions in this dataset. The Garmin wrist layer (incorporating heart rate, skin temperature, and wrist kinematics) contributes the second-highest mean per feature. HRV features contribute moderately, and the smaller magnitude reflects that HRV signal in 60-s free-living windows is inherently weaker than in longer windows or controlled exposures. OPPO kinematic features contribute at a similar level per feature, and magnetometer magnitude features contribute the least but remain non-trivial. All five data layers contribute meaningfully to predictions, with no single layer dominating to the exclusion of others. The implications of these layer contributions are discussed in Section 5.

## 5. Discussion

This section discusses the key findings of the proposed framework, focusing on feature interpretation, model robustness, and real-world applicability, while highlighting limitations and comparisons with existing work.

### 5.1. Feature Interpretation

The SHAP analysis reveals a feature distribution that aligns with established physiological and environmental comfort literature. The top 10 features span four data layers and capture genuine comfort-relevant signals across acoustic, thermal, and physiological domains.

Acoustic features dominate the top 10, with sound_sd, sound_mean, and sound_min all ranking in the top four positions and sound_max ranking nearby. This is consistent with the established literature on acoustic stress: El Aarbaoui et al. [6] demonstrated measurable autonomic responses to noise at levels as low as 40 dB(A) in free-living conditions, and Zhen et al. [19] established that acoustic exposure interacts nonlinearly with thermal stress to drive overall discomfort. The retention of acoustic features in absolute form rather than within-session normalised, justified physically by the consistent smartphone deployment across all sessions, allows the model to learn the genuine relationship between acoustic exposure level and comfort state across environments.

Four HRV features (rmssd, sd1, sdsd, hf_power) appear in the top 10. This is consistent with established physiology: rmssd, sd1, and sdsd are mathematically related short-term variability measures reflecting parasympathetic activity, and hf_power reflects parasympathetic vagal tone entrained by respiratory sinus arrhythmia. Their appearance confirms that ECG-derived HRV signal carries comfort-relevant information independently of activity, even though the absolute SHAP magnitudes are modest. The relatively small per-feature contribution of HRV reflects a fundamental constraint of free-living HRV measurement at 60-s window resolution: HRV signal-to-noise ratio is substantially lower than in longer windows or controlled exposures, where significant sustained autonomic shifts produce stronger feature variation.

The thermal feature temp_mean ranks fifth, demonstrating that wrist skin temperature mean is a meaningful comfort signal. Wrist temperature integrates the body’s thermal response across thermal environment components without requiring direct measurement of operative temperature, providing a physiologically grounded signal in genuine free-living conditions.

Two kinematic context features appear in the top 10 (wrist_accel_mag_mean at rank 1 and ay_sd at rank 2). These features capture activity level rather than direct environmental comfort signals. Their high SHAP contribution reflects the activity–comfort correlation in the dataset rather than a fundamental physiological relationship, and is interpreted in detail in Section 5.2.

### 5.2. Activity–Comfort Confound

Two of the top ten SHAP features (wrist_accel_mag_mean at rank 1 and ay_sd at rank 2) capture physical activity level rather than direct environmental comfort signals. Despite excluding activity-correlated proxies such as phone gravity components and per-axis magnetometer values from the feature set, kinematic features remain prominent in the model’s decision-making.

This persistence reflects two structural characteristics of the dataset. First, activity level is correlated with comfort state at the dataset level in this pilot, with mean phone accelerometer magnitude approximately twice as high in uncomfortable windows (0.78 m/s^2^) as in comfortable windows (0.42 m/s^2^) as shown in Table 3. This dataset-level correlation is driven primarily by a small number of high-movement sessions, including outdoor walking routes and rapid transit through busy public spaces, that were predominantly labelled uncomfortable due to environmental conditions encountered during the activity, rather than by a within-session coupling between movement and discomfort. Within sessions where both comfort classes occurred, the activity–comfort relationship is weak or in some cases reversed: in several sedentary indoor sessions, the comfortable windows show higher mean accelerometer magnitude than the uncomfortable windows. The model picks up the dataset-level correlation as a partial comfort signal even though the relationship is incidental to the underlying physiology. Second, kinematic features serve a legitimate role as activity context, allowing the model to interpret physiological signals against the backdrop of physical movement. The challenge is that activity context and activity confound are not cleanly separable in observational free-living data.

A subset of new sessions in this pilot were conducted in sedentary settings where the author remained seated throughout while thermal and acoustic conditions changed naturally within the session. These sessions provide the model with comfort transitions that are not coupled to changes in activity, weakening the activity–comfort correlation in the training data without departing from genuine free-living conditions. The continued top ranking of kinematic features after these sessions were added indicates that activity correlation persists across the broader dataset, but the confound is reduced compared to a dataset comprising only naturalistic activity-varying sessions.

Fully resolving the activity–comfort confound requires further data collection beyond what this pilot achieves. Future deployments should ensure that comfortable and uncomfortable sessions span comparable activity levels, for example pairing a sedentary uncomfortable session in a thermally stressful environment with a sedentary comfortable session in a thermally neutral environment. Without such balance across the dataset, classifiers risk learning activity patterns that correlate with comfort state in the training distribution but do not generalise to deployments where this correlation does not hold.

### 5.3. Macro-Environmental Signal Exclusion

All macro-environmental variables retrieved from the Open-Meteo API, including temperature, humidity, air quality, and related variables, were excluded from the modelling feature set. Analysis showed that these variables varied primarily between sessions rather than within sessions, acting as session identity proxies rather than capturing within-session comfort dynamics. Across 18 sessions collected in Melbourne under broadly similar autumn conditions, the macro-environmental range was insufficient to contribute predictive signal at the 60-s window level.

This is a fundamental constraint of retrospective API-based environmental data: hourly resolved meteorological values cannot capture the micro-level environmental variation that drives within-session comfort changes. A participant moving between a sun-exposed outdoor space and a shaded area within the same session experiences a meaningful thermal change that an hourly API value cannot resolve. Air quality variables face the same constraint with an additional limitation: regional outdoor air quality estimates do not reflect localised indoor conditions including CO_2_ accumulation, particulate matter, volatile organic compounds, and odours, which can substantially affect indoor occupant comfort but are not measurable from regional API data.

Addressing these limitations in future deployments requires personal environmental sensing rather than regional API data. A wearable air quality monitor such as the Atmotube Pro, which measures VOCs, PM_1_, PM_2.5_, PM_10_, temperature and humidity in a compact form factor consistent with the framework’s consumer-grade hardware design principle, would integrate into the existing pipeline as an additional data layer without requiring modifications to the feature extraction or modelling architecture. Alternatively, deployment across a wider range of seasons and geographic locations would create sufficient between-session variation in macro-environmental conditions to contribute predictive signals at the between-session level.

### 5.4. Comparison with Related Work

Direct comparison of classification performance with prior work requires careful contextualisation, as study conditions vary substantially. Chaudhuri et al. [10] reported approximately 97% accuracy for thermal comfort prediction in a controlled climate chamber using wrist pulse rate and skin temperature. Kim et al. [11] achieved 79.7% accuracy with LightGBM in an indoor office setting. Both studies operate in controlled, single-domain environments with steady-state conditions and no activity confounds, conditions fundamentally more favourable to classification than the unconstrained free-living deployment presented here.

The most directly comparable work is Mishra et al. [15], who reported an F1 of 0.502 with physiological features alone and 0.769 with contextual features added in a free-living stress detection study with 23 participants. The Random Forest F1 macro of 0.456±0.151 achieved in this pilot is modestly lower than Mishra’s physiology-only baseline.

Dai et al. [20], the most complete existing multi-domain study, demonstrated that individual models consistently outperform general models in office environments, a finding that directly motivates the personalised modelling approach taken here. Their study, however, remained confined to indoor office settings without addressing free-living transitions across multiple environment types. The performance achieved in the present pilot, while modest, reflects the difficulty of extending physiological comfort prediction beyond controlled or semi-controlled settings into genuinely unconstrained conditions.

Across these comparisons, a consistent pattern emerges: classification performance degrades as the experimental setting moves from controlled chamber to controlled office to free-living deployment. The pilot results presented here represent the lower-performance end of this spectrum, and characterise empirically what consumer wearable comfort sensing can and cannot achieve under realistic free-living conditions. The findings inform expectations and design choices for future research seeking to deploy such systems outside laboratory environments.

### 5.5. Limitations

Several aspects of this pilot constrain the strength of the conclusions that can be drawn and merit honest characterisation.

The dataset comprises 18 sessions from a single author, of which 10 produced valid LOSO test folds. Although this is a substantial expansion from initial pilot data, it remains insufficient to support strong generalisation claims, and the large standard deviations across folds (Random Forest F1 macro 0.456±0.151) reflect genuine uncertainty from limited data and variable session sizes rather than measurement noise. These results should be interpreted as proof of concept for the pipeline and an honest characterisation of free-living comfort sensing performance, rather than as reliable estimates of deployed system performance at scale.

Physiological signals in free-living conditions are influenced by factors beyond environmental comfort. Physical activity remains a partially unresolved confound despite feature exclusions and the inclusion of sedentary sessions, as discussed in Section 5.2. Other factors including fatigue, psychological stress, recent food or caffeine intake, and sleep quality affect autonomic measurements and were not controlled in this pilot. HRV signal-to-noise at the 60-s window resolution used here is constrained by the ultra-short-term HRV regime: while RMSSD and pNN50 retain reasonable reliability at this resolution, LF power and SDNN are known to be substantially less stable than at the 5-min short-term standard [24,25]. The modest contribution of HRV features in SHAP analysis reflects this constraint in addition to the structural challenge of detecting subtle autonomic shifts under naturalistic comfort transitions, and may understate the true comfort-relevance of HRV signal that longer windows would surface. The lap-button labelling protocol introduces temporal uncertainty near state transitions. Comfort state changes are gradual subjective processes rather than discrete events with objective timestamps, meaning windows that span a labelled transition carry a label corresponding to either the pre-transition or post-transition state when in physiological terms they likely contain a mixture of both. This is a known constraint of ecological momentary assessment approaches [15], and the magnitude of its effect on classification performance is not characterised in this pilot. Future deployments could implement a transition buffer that excludes windows within a fixed interval of each lap press from training and evaluation, allowing the sensitivity of classification performance to label-edge noise to be quantified directly.

The framework captures only a subset of the four IEQ domains it is architecturally designed to address. Visual sensing was not achieved due to the deliberate decision to maintain consistent smartphone deployment in the trouser pocket, with the luxometer measuring pocket openness rather than ambient light. Localised indoor air quality including CO_2_, particulates, and volatile organic compounds was not measured as the framework relied on regional API estimates that do not resolve indoor conditions. Both gaps are addressable through additional hardware (a wrist-worn or chest-mounted light sensor, and a wearable air quality monitor such as the Atmotube Pro) without requiring changes to the feature extraction or modelling architecture.

Manual identification of synchronisation points across the OPPO and Garmin streams remains a practical scaling limitation. Three automated approaches were evaluated and found unreliable across diverse session conditions, and manual identification at approximately two minutes per session was adopted as the most consistent method for this pilot. Larger deployments will require a validated automated synchronisation method.

## 6. Conclusions

This paper proposed the Comfort Framework, a multimodal data fusion system for real-world personalised comfort prediction using consumer wearables. The framework is architecturally designed to address all four major IEQ domains of thermal, acoustic, visual, and air quality, with a complete pipeline spanning multi-device data collection, synchronisation, feature extraction across five sensor modalities, and binary comfort classification with SHAP-based interpretability.

A single-subject pilot was conducted across 18 free-living sessions in Melbourne, producing 418 labelled 60-s windows. Of the 18 sessions, 10 yielded valid LOSO cross-validation folds, with the remaining 8 single-class sessions contributing to training but not evaluation. Random Forest achieved the highest classification performance with F1 macro of 0.456±0.151 across the valid folds. This performance is modest, situating at the lower end of the chamber-to-office-to-free-living spectrum reported in prior comfort and stress detection literature, and reflects the genuine difficulty of physiological comfort prediction under unconstrained naturalistic conditions.

After excluding features that captured session and location identity rather than comfort signal—including altitude, phone gravity components, and per-axis magnetometer values—the model relies on physically interpretable comfort signals: acoustic exposure, HRV, wrist skin temperature, and kinematic context. SHAP analysis on this feature set showed that acoustic features contribute the strongest signal per feature, followed by Garmin wrist measurements (heart rate, temperature, wrist movement), HRV features, OPPO kinematic features, and magnetometer magnitude features.

The pilot validates the pipeline architecture and characterises empirically what consumer wearable comfort prediction can and cannot achieve under genuine free-living conditions. Of the four IEQ domains, thermal and acoustic signals were captured and contributed to classification, while visual and localised indoor air quality were not validated due to deployment and instrumentation constraints.

Two structural limitations of observational free-living data shape the pilot’s results. The activity–comfort coupling at the dataset level, while partially weakened through feature selection and the inclusion of sedentary sessions, was not eliminated, as a within-session decoupling of activity from comfort would require deliberate experimental design across sessions. The narrow geographic and seasonal range of the Melbourne pilot prevented macro-environmental variables from contributing within-session predictive signal at the feature level, a constraint that wider-deployment data collection would address directly.

Three directions are identified for future work. First, larger cohorts with cross-subject diversity are required to assess generalisability and support personalised modelling at scale. Second, additional hardware including a wrist-worn or chest-mounted light sensor and a wearable air quality monitor would extend domain coverage to visual and indoor air quality without modifying the pipeline architecture. Third, future deployments should ensure that comfortable and uncomfortable sessions span comparable activity levels to reduce the activity–comfort correlation in training data.

The framework and pipeline are designed to be practically deployable with consumer-grade hardware: a chest strap, a smartwatch, and a smartphone, without requiring specialised environmental monitoring equipment. The pilot establishes both the feasibility of the approach and the realistic performance envelope to expect under free-living conditions, providing a foundation for further methodological development and larger-scale validation.

## Figures and Tables

**Table 1:** Feature set by data layer. A total of 55 features are used in modelling.

Layer	Source	Features
HRV time domain	Polar H10 via Garmin	mean_rr, sdnn, rmssd, sdsd, pnn50, mean_hr
HRV frequency domain	Polar H10 via Garmin	lf_power, hf_power, lf_hf_ratio, total_power
HRV nonlinear	Polar H10 via Garmin	sd1, sd2, sd1_sd2_ratio, sample_entropy
HR and reserve	Garmin 1 Hz	hr_mean, hr_sd, hr_min, hr_max, hr_reserve_mean
Wrist temperature	Garmin	temp_mean, temp_sd, temp_rate_of_change
Wrist kinematics	Garmin 25 Hz	wrist_accel_mag_mean, wrist_accel_mag_sd, wrist_accel_mag_max
Kinematic	OPPO accelerometer	accel_mag (mean, sd, min, max), per-axis accelerometer means and standard deviations, jerk (mean, sd, max)
Kinematic	OPPO gyroscope	gyro_mag (mean, sd, max), per-axis gyroscope means and standard deviations
Acoustic	OPPO microphone	sound_mean, sound_sd, sound_min, sound_max
Magnetic field	OPPO magnetometer	mag_mag_mean, mag_mag_sd, mag_mag_min, mag_mag_max
Excluded	Various	Macro-environmental variables (varied between sessions only); light features (pocket deployment artifact); altitude, gravity components, per-axis magnetometer values, heading variance (location and posture identity proxies); n_beats, gyro_mag_min, pressure features, gps_speed_mean, contextual session proxies (technical limitations). See Section 3 for justification.

**Table 2:** Dataset summary across 18 free-living sessions.

Property	Value
Total sessions	18
Total windows	418
Comfortable windows	223 (53.3%)
Uncomfortable windows	195 (46.7%)
Single-class sessions (skipped)	8
Valid LOSO folds	10
Window length	60 s (non-overlapping)
Environments captured:	
Indoor home, warehouse, library, café, train, indoor public spaces	
Outdoor: walking routes, urban environments	

**Table 3:** Descriptive statistics for selected features by comfort state across the 418 labelled windows. Values are mean ± SD with [min, max] in brackets. Sound levels are reported in absolute decibel form as recorded by the smartphone microphone in consistent pocket deployment ^1^; physiological, thermal, and kinematic values are reported in their original units prior to within-session normalisation. Window counts differ from class totals because HRV-only features (rmssd, hf_power) include windows where Garmin record streams were unavailable.

Feature	Comfortable	Uncomfortable
Wrist temperature (°C)	31.9 ± 1.7 [27.5, 36.0]	33.3 ± 2.5 [28.0, 37.0]
Heart rate (bpm)	70.7 ± 7.6 [51.8, 90.2]	77.1 ± 6.7 [63.7, 93.0]
RMSSD (ms)	42.1 ± 20.4 [9.5, 129.1]	34.3 ± 22.3 [9.9, 164.9]
HF power (ms^2^)	12.3 ± 11.9 [0.2, 66.3]	8.5 ± 11.2 [0.3, 89.6]
Sound mean (dB)	78.0 ± 9.3 [51.6, 96.2]	77.4 ± 12.9 [48.4, 100.3]
Sound SD (dB)	5.9 ± 3.0 [1.0, 14.3]	4.8 ± 2.5 [0.5, 11.2]
Phone accel magnitude (m/s^2^)	0.42 ± 0.88 [0.06, 11.00]	0.78 ± 2.25 [0.05, 15.22]

^1^ Smartphone microphone dB readings are reported as recorded by the Physics Toolbox Sensor Suite and represent a consistent relative measure across sessions rather than ISO-calibrated sound pressure level. The consistency of pocket deployment makes between-session comparison meaningful regardless of absolute calibration offset.

**Table 4:** Leave-one-session-out cross-validation results across 10 valid folds (mean ± std). Best result per metric in bold.

Classifier	F1 Macro	F1 Uncomf.	Precision	Recall	AUC
Naive Bayes	0.374 ± 0.127	0.424 ± 0.212	0.451 ± 0.122	0.435 ± 0.123	0.394 ± 0.144
SVM (RBF)	0.437 ± 0.176	0.453 ± 0.260	0.490 ± 0.161	0.481 ± 0.197	0.502 ± 0.204
**Random Forest**	**0.456 ± 0.151**	0.450 ± 0.180	**0.525 ± 0.118**	**0.527 ± 0.163**	**0.519 ± 0.163**
XGBoost	0.414 ± 0.143	**0.462 ± 0.164**	0.455 ± 0.145	0.487 ± 0.120	0.482 ± 0.189

**Table 5:** Top 10 features by mean absolute SHAP value (Random Forest). Higher values indicate greater contribution to individual predictions.

Rank	Feature	Physical Meaning	Mean |SHAP|
1	wrist_accel_mag_mean	Mean wrist movement—activity context	0.0301
2	ay_sd	Phone accelerometer *Y*-axis variability—activity context	0.0233
3	sound_sd	Acoustic exposure variability—ambient noise fluctuation	0.0208
4	sound_mean	Mean acoustic exposure level—ambient noise	0.0177
5	temp_mean	Mean wrist skin temperature—thermal state	0.0164
6	rmssd	Root mean square of successive RR differences—parasympathetic activity	0.0162
7	sd1	Poincaré short-term variability—short-term HRV	0.0153
8	sdsd	Standard deviation of successive RR differences—short-term HRV	0.0153
9	hf_power	HF spectral power—parasympathetic vagal tone	0.0147
10	sound_min	Minimum acoustic exposure—quiet floor of environment	0.0140

**Table 6:** Mean absolute SHAP value aggregated by data layer across the 55 features used in modelling.

Data Layer	Features (n)	Mean |SHAP| per Feature
Acoustic	4	0.01535
HR & wrist (Garmin)	11	0.00930
HRV (physiological)	14	0.00771
Kinematic (OPPO)	22	0.00760
Magnetometer (magnitude)	4	0.00731

## Data Availability

The data presented in this study are available on request from the corresponding author.

## References

[1] Al Horr Y., Arif M., Katafygiotou M., Mazroei A., Kaushik A.K., Elsarrag E. (2016). Impact of indoor environmental quality on occupant well-being and comfort: A review of the literature. Int. J. Sustain. Built Environ..

[2] Sundstrom E., Town J.P., Rice R.W., Osborn D.P., Brill M. (1994). Office Noise, Satisfaction, and Performance. Environ. Behav..

[3] Pan L., Lian Z., Lan L. (2012). Investigation of sleep quality under different temperatures based on subjective and physiological measurements. HVAC&R Res..

[4] Frontczak M., Wargocki P. (2011). Literature survey on how different factors influence human comfort in indoor environments. Build. Environ..

[5] Liu W., Lian Z., Liu Y. (2008). Heart rate variability at different thermal comfort levels. Eur. J. Appl. Physiol..

[6] El Aarbaoui T., Méline J., Brondeel R., Chaix B. (2017). Short-term association between personal exposure to noise and heart rate variability: The RECORD MultiSensor Study. Environ. Pollut..

[7] Fanger P.O. (1970). Thermal Comfort: Analysis and Applications in Environmental Engineering.

[8] Yang C., Taniguchi K., Akashi Y. (2025). Multi-source domain adaptation for personalized thermal comfort prediction using wearable sensors. Energy Build..

[9] Kim J., Schiavon S., Brager G. (2018). Personal comfort models-A new paradigm in thermal comfort for occupant-centric environmental control. Build. Environ..

[10] Chaudhuri T., Soh Y.C., Li H., Xie L. (2020). Machine learning driven personal comfort prediction by wearable sensing of pulse rate and skin temperature. Build. Environ..

[11] Kim H., Lee G., Ahn H., Choi B. (2024). Interpretable general thermal comfort model based on physiological data from wearable bio sensors: LightGBM and SHAP. Build. Environ..

[12] Petrowski K., Mekschrat L., Bührer S., Siepmann M., Albus C., Schmalbach B. (2023). Effects of Post-awakening Light Exposure on Heart Rate Variability in Healthy Male Individuals. Appl. Psychophysiol. Biofeedback.

[13] Cakmak S., Kauri L., Shutt R., Liu L., Green M.S., Mulholland M., Stieb D., Dales R. (2014). The association between ambient air quality and cardiac rate and rhythm in ambulatory subjects. Environ. Int..

[14] Nikolopoulou M., Steemers K. (2003). Thermal comfort and psychological adaptation as a blind spot in urban design. Energy Build..

[15] Mishra V., Hao T., Sun S., Walter K.N., Ball M.J., Chen C.H., Zhu X. (2018). Investigating the Role of Context in Perceived Stress Detection in the Wild. Proceedings of the 2018 ACM International Joint Conference on Pervasive and Ubiquitous Computing and the 2018 International Symposium on Wearable Computers.

[16] Aqajari S.A.H., Labbaf S., Tran P.H., Nguyen B., Mehrabadi M.A., Levorato M., Dutt N., Rahmani A.M. (2024). Context-Aware Stress Monitoring using Wearable and Mobile Technologies in Everyday Settings. arXiv.

[17] Huang L., Zhu Y., Ouyang Q., Cao B. (2012). A study on the effects of thermal, luminous, and acoustic environments on indoor environmental comfort in offices. Build. Environ..

[18] Buratti C., Belloni E., Merli F., Ricciardi P. (2018). A new index combining thermal, acoustic, and visual comfort of moderate environments in temperate climates. Build. Environ..

[19] Zhen M., Chen Z., Zou Q. (2023). Combined effects of thermal and acoustic environments on outdoor human comfort in university campus. Urban Clim..

[20] Dai H., Imani S., Choi J.H. (2025). Correlating Indoor Environmental Quality Parameters with Human Physiological Responses for Adaptive Comfort Control in Commercial Buildings. Energies.

[21] Ibrahim I.I., Shukor S.A.A., Rahim M.A. Heart Rate as a Physiological Indicator of Discomfort. Proceedings of the 2025 30th International Conference on Automation and Computing (ICAC).

[22] Sahoh B., Wongsontham F., Tipsavak A., Chaithong P., Kliangkhlao M., Efendi M.A., Songnuy T., Punsawad Y. (2025). Deep learning-based comparative evaluation of EEG, HRV, and EDA biomarkers for personal thermal comfort prediction. Measurement.

[23] Task Force of the European Society of Cardiology and the North American Society of Pacing and Electrophysiology (1996). Heart rate variability: Standards of measurement, physiological interpretation, and clinical use. Circulation.

[24] Shaffer F., Ginsberg J.P. (2017). An Overview of Heart Rate Variability Metrics and Norms. Front. Public Health.

[25] Muñoz M.L., van Roon A., Riese H., Thio C., Oostenbroek E., Westrik I., de Geus E.J.C., Gansevoort R., Lefrandt J., Nolte I.M. (2015). Validity of (Ultra-)Short Recordings for Heart Rate Variability Measurements. PLoS ONE.

[26] Richman J.S., Moorman J.R. (2000). Physiological time-series analysis using approximate entropy and sample entropy. Am. J. Physiol. Heart Circ. Physiol..

